# Survey-Based Insights into Romania’s Pathology Services: Charting the Path for Future Progress

**DOI:** 10.3390/healthcare13111302

**Published:** 2025-05-30

**Authors:** Maria Magdalena Köteles, Ovidiu Țica, Gheorghe Emilian Olteanu

**Affiliations:** 1 Pathology Department, County Emergency Hospital Bihor, 410169 Oradea, Romania; 2 Department of Morphological Disciplines, Faculty of Medicine and Pharmacy, University of Oradea, 410073 Oradea, Romania; 3 Department of Pathology, British Columbia Cancer Agency, Vancouver, BC V5Z4E6, Canada; emilian.olteanu@bccancer.bc.ca; 4 Department of Pathology and Laboratory Medicine, University of British Columbia, Vancouver, BC V6T 1Z7, Canada

**Keywords:** pathology laboratories, public healthcare system, infrastructure, patient care, Romania

## Abstract

Background: Pathology is essential for cancer diagnosis, bridging clinical and surgical fields, and requires adequate infrastructure, technology, and skilled staff to meet standards of care. In Romania, healthcare underfunding limits pathology laboratories’ capacity to provide timely and accurate diagnoses, leading to delays that could negatively impact treatment and patient outcomes. Our study aimed to assess the status of publicly funded pathology laboratories in Romania and identify key areas for improvement. Methods: We analyzed public hospitals in Romania, excluding specialized and non-general care institutions, to evaluate pathology laboratories. A 10-item survey was distributed over 12 months via email, phone, administrative offices, and professional networks to pathologists working in these laboratories, regardless of their hierarchical position. A total of 154 pathology services were represented. The questionnaire assessed technical capabilities, diagnostic techniques, automation, staffing, infrastructure, and satisfaction with funding and resources. Responses were gathered with both predefined and open-text fields to capture comprehensive insights. Results: The findings revealed that many pathology laboratories faced significant challenges, including a lack of automation, limited integration of modern technologies, and barriers to digitalization. Despite these issues, pathologists reported higher-than-expected levels of satisfaction with their laboratories. Conclusions: A comprehensive understanding of existing practices is necessary to drive the modernization of pathology services, establish national standards, and improve collaboration both within and across specialties. Without such foundational insight, efforts to enhance the integration and effectiveness of pathology services are likely to remain constrained.

## 1. Introduction

Pathology serves as a vital intermediary between clinicians and surgeons, playing an essential role in the diagnostic process. As such, pathology laboratories (PLs) are fundamental to the efficient functioning of any healthcare system (HS) [1]. As the field of pathology evolves with advancements in molecular classification and immunotherapy, PLs must assess their current capabilities to align with emerging standards. It is essential for modern histopathologists to be proficient in traditional techniques while adapting to new technologies. Since implementing changes often requires specific accreditations, a thorough baseline assessment is a necessary first step before modernization and standardization efforts can be undertaken. The White Book of Pathology in Spain (2023) illustrates how collecting and publishing structured national data can support this process [2]. By highlighting variability in pathology services and establishing performance benchmarks, it helps guide quality improvement and policy alignment. Across Europe, pathology laboratories still show considerable variation, with inconsistencies often reflecting broader differences in national health priorities and the transparency of available data. In contrast, countries without such centralized evaluations may struggle to identify gaps or justify the need for investment, ultimately slowing progress toward harmonization and accreditation [3]. 

Romania’s public HS has faced numerous challenges in recent years, yet it has continued to provide patient care with limited resources. PLs are no exception. Outdated legislation [4], insufficient funding, and a lack of standardization are among the issues that hinder the integration of both basic and advanced diagnostic techniques in public PLs. The consistently high patient volume, paired with ongoing scientific advancements, such as the integration of Artificial Intelligence (AI), genomic analysis, and molecular profiling, place significant strain on PLs and pathologists, demanding resources that are often unavailable [5,6,7]. 

Patients are the ones who bear the burden of inadequate healthcare infrastructure, particularly when laboratories lack the resources to offer personalized medicine and high-quality care. While some countries do not face an overall shortage of doctors, studies have shown that certain specialties, such as pathology, experience a significant gap in demand [8,9]. The number of pathologists per 100,000 inhabitants is relatively low in many regions of Europe, and this shortage is especially noticeable in eastern countries like Romania [10]. Compounding this issue is the outdated curriculum for pathology trainees, which, in Romania, has not been updated since 2017 [11]. As a result, new generations of pathologists may struggle to acquire the latest diagnostic techniques and technological advancements, further limiting the quality of care patients receive. The shortage of specialists, coupled with insufficient training and underdeveloped laboratory infrastructure, creates significant barriers to timely and accurate diagnoses. Consequently, patients’ healthcare journeys are made more difficult and prolonged, placing added pressure on an already overstretched HS. The situation highlights the urgent need for systemic reforms, not only to increase the number of specialists, but also to modernize educational curricula and upgrade infrastructure to keep pace with the rapidly evolving patient care.

Our study aimed to evaluate the current state of PLs within Romania’s public HS, assessing for the first time a range of technical and organizational aspects that can represent a starting point to national standardization.

## 2. Materials and Methods

Using the publicly available Ministry of Health’s website, we identified a classification of both public and private hospitals across the entire territory of Romania for the year 2023 [12]. For the purposes of our analysis, we specifically focused on public hospitals, excluding psychiatric institutions, palliative care, and hospice centers, as well as balneary, rehabilitation, sanatorium hospitals, and penitentiary healthcare facilities. We then conducted a detailed review of the websites of the remaining hospitals to ascertain the information available for PLs.

To assess the current practices within these PLs, we developed a 10-item questionnaire designed to fully align with the Romanian context, a tailored instrument reflecting local realities and needs in order to gather comprehensive data on various aspects of laboratory operations (Supplementary Materials). The survey was distributed over a 12-month period using multiple communication channels, beginning with email. Specifically, 36 laboratories provided email addresses through the hospital’s website, while an additional 118 hospitals were contacted via their administrative offices. The remaining hospitals were reached by phone to obtain relevant email contacts. Furthermore, the National Society of Pathology’s (Societatea Profesională Națională Română “Uniunea Patologilor”) official WhatsApp group facilitated additional responses, and a reminder, sent via mail with an acknowledgment of receipt form to confirm delivery, was sent to the 106 non-responding hospitals. Participation in the survey was open to all individuals employed within the PL, regardless of their role or hierarchical position. The survey was conducted anonymously. All participants were informed of the study’s purpose, and their completion of the survey was taken as consent to participate. Online responses collected via Google Forms did not log IP addresses or require personal identifiers linked to the responses. For responses received via email or WhatsApp, all identifying information of the individual respondent was removed before data analysis to ensure confidentiality. Data are presented in an aggregated, descriptive statistical format to prevent the identification of individual respondents or institutions.

The questionnaire was developed by the research team, to gather comprehensive data and explore key aspects of PL practices. The questions were formulated to assess the technical capabilities of the laboratories, the extent of automation and digitalization, available diagnostic equipment and techniques, e.g., immunohistochemistry (IHC), fluorescence in situ hybridization (FISH), chromogenic in situ hybridization (CISH), immunofluorescence (IF), next-generation sequencing (NGS), the number of specialized pathologists employed, and the status of laboratory renovations and infrastructure improvements. A primary guiding document for assessing technical capabilities was the “List of Mandatory Minimum Equipment for Pathological Anatomy and Forensic Services” issued by the Romanian Ministry of Health, which served as a benchmark for expected standards [4]. The questions were informed by common themes in the pathology service evaluation literature and tailored to the specific context of the Romanian public HS. The aim was to create a concise yet informative survey that could be completed in a reasonable timeframe to encourage participation.

For most questions, respondents selected answers from a predefined list of options. A free-text field was provided for respondents to suggest additional diagnostic techniques they believed should be incorporated into the PL. Additionally, two questions were included to evaluate participants’ satisfaction with the current state of laboratory infrastructure and funding levels, using a 1 to 10 scale to offer more nuanced distinctions in respondents’ assessments, where 10 indicated “very satisfied” and 1 indicated “not satisfied at all”. 

All the data collected during the survey are presented as descriptive statistics.

## 3. Results

Partial data from this study were presented as a physical scientific poster (Survey-based evaluation of pathology services within Romania’s healthcare system) during the 36th European Congress of Pathology held in Florence, Italy, from 7 to 11 September 2024. 

Out of the 459 hospitals identified and listed under the coordination of the Romanian Ministry of Health, and distributed across 41 counties and the Municipality of Bucharest, a total of 338 public hospitals were included in our analysis after applying the exclusion criteria. Among these 338 hospitals, 159 were found to have active pathology laboratories at the time of data collection.

Out of the identified 159 PLs, 85 (53.46%) responded to our request, and 80 (50.31%) completed the questionnaire. Of the five respondents who did not complete the survey, three laboratories declined the invitation, one was outsourced, and one was in the process of relocation. Responses were received through various channels: 40% via Google Forms, 32.5% through direct email or WhatsApp, and an additional 27.5% following the dispatch of mail.

### 3.1. Technical Capabilities

The technical infrastructure of the responding laboratories was compared to the “List of Mandatory Minimum Equipment for Pathological Anatomy and Forensic Services” provided by the Ministry of Health in the Romanian Legislative Publication (“Monitorul Oficial”) [4] (Table 1).

**Figure 1:**
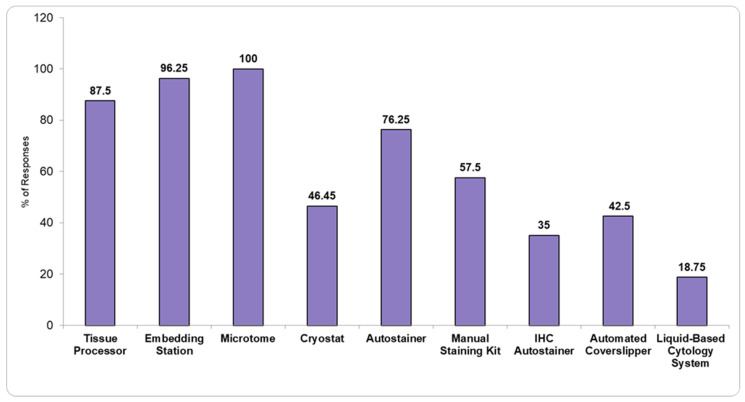
Available equipment in pathology laboratories.

**Figure 2:**
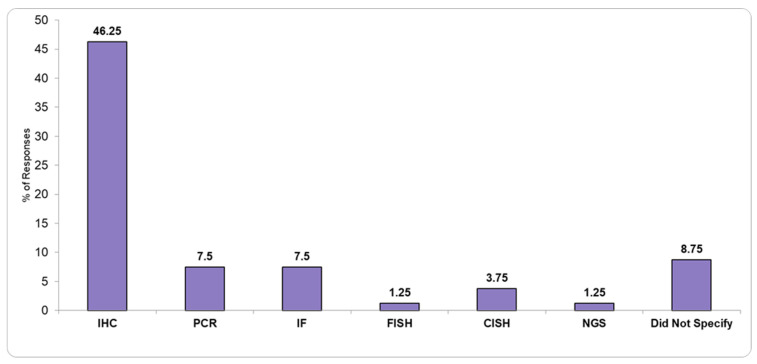
Available diagnostic techniques in pathology laboratories.

Figure 1 and Figure 2 show the available basic pathology equipment, and the available diagnostic techniques present in the PL î that responded to the questionnaire.

**Figure 3:**
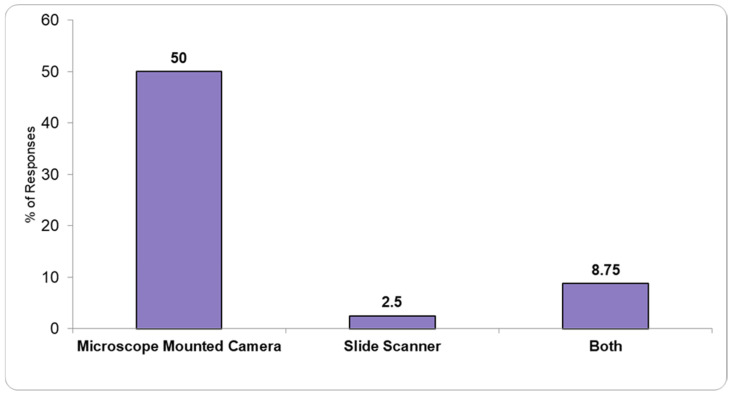
Available digital microscopy device in pathology laboratories.

A total of 61.25% of the responding PLs confirmed they had the necessary infrastructure to obtain digital slides (Figure 3). Half of the responders mentioned the use of microscope cameras, 2.5% slide scanners, while 8.75% stated having both capabilities.

### 3.2. Pathologist Employment

The survey also included a section for respondents to report the number of pathologists employed in their laboratories. Out of the total 80 responding hospitals, data analysis revealed an average of 3.6 pathologists per PL.

### 3.3. Laboratory Renovations and Infrastructure Improvements

**Figure 4:**
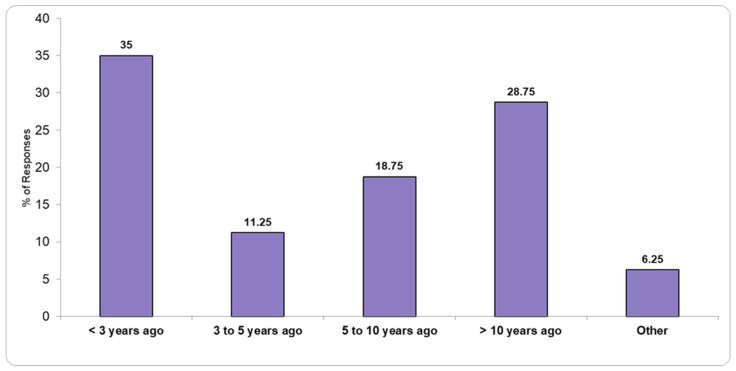
Renovation status of the pathology laboratory.

**Figure 5:**
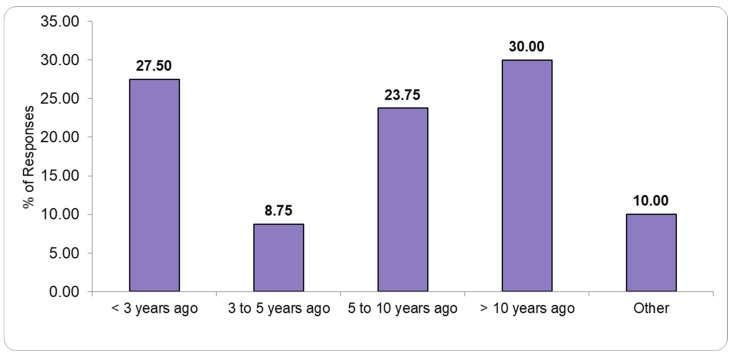
Renovation status of the morgue in pathology laboratories.

Figure 4 and Figure 5 illustrate the status of recent laboratory renovations and infrastructure improvements, with responses indicating various levels of modernization and ongoing development efforts.

### 3.4. Satisfaction with Infrastructure and Funding

**Figure 6:**
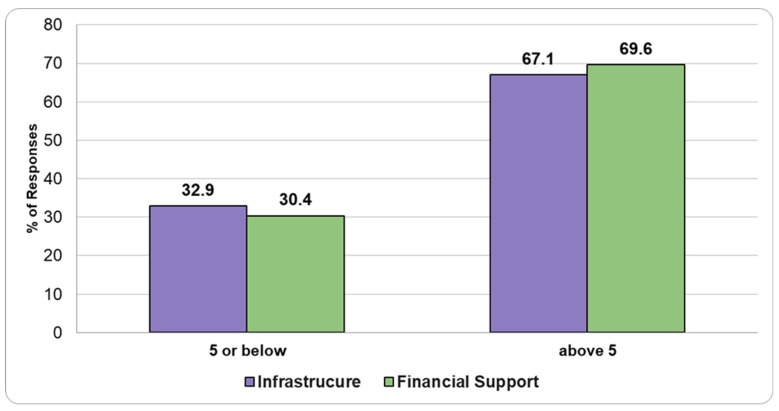
Satisfaction levels.

Regarding satisfaction with infrastructure and financial support, respondents rated their satisfaction on a scale from 1 to 10, where 1 indicated “not satisfied at all” and 10 indicated “very satisfied”. Approximately 30% of respondents rated their satisfaction at 5 or lower, while around 70% rated it above 5 for both infrastructure and financial support (Figure 6).

### 3.5. Free-Text Responses and Suggested Improvements

**Figure 7:**
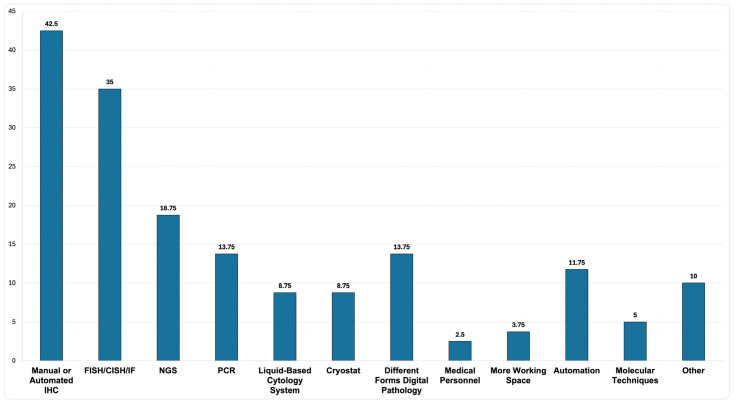
Suggested improvements from respondents. Categorized responses from the survey participants regarding suggested improvements. Responses were grouped into categories based on keywords identified in the free-text answers. The figure shows the number of respondents who suggested each category and the corresponding percentage of the total respondents.

Analysis of the free-text responses revealed that over 40% of PLs expressed a need for manual or automated IHC staining, among other suggested improvements related to advanced diagnostic techniques, equipment, and enhancements to personnel and workspace (Figure 7).

## 4. Discussion

The comparison of healthcare spending per capita in Romania with Western European countries such as Germany highlights notable disparities driven by various economic and social factors. As one of the European Union’s (EU) lower Gross Domestic Product (GDP) nations, Romania spends considerably less on healthcare per capita compared to other Western European countries [13,14]. Statistics show that countries with lower GDPs, such as Romania or Bulgaria, face higher relative costs for healthcare services, yet their overall healthcare spending remains much lower than that of higher GDP countries [14,15]. 

The classification of financial mechanisms in EU countries further highlights that Romania’s healthcare spending per capita is significantly lower than that of more developed EU nations, reflecting the broader economic challenges faced by post-socialist countries. Additionally, it has been noted that Romania’s initial government expenditure on healthcare per capita was much lower than that of well-developed European countries [16]. This historical underfunding has had lasting effects on Romania’s HS, where public health expenditures continue to fall short of meeting the population’s needs [17]. 

Our study shows that the available equipment in the included PL is generally rudimentary. As such, 23.75% and 65% do not use automated Hematoxylin and Eosin (H&E) or IHC stainers, respectively, limiting standardization and the potential for advanced diagnostic capabilities. These laboratories lack the infrastructure necessary for digitalization and automation of workflows, which are essential for enhancing diagnostic efficiency and accuracy. Despite the existence of an official list of mandatory minimum equipment issued by the Ministry of Health [4], some hospitals fail to meet even these basic requirements (Figure 1). This raises concerns about whether the standards outlined in the list are overly ambitious and unachievable, or whether the laboratories themselves need to significantly improve their operational capacity to meet these expectations.

We also found that nearly 40% of PLs in Romania lack both slide scanners and camera attachments for microscopes, significantly hindering the transition to digital pathology (DP). The absence of these essential tools limits the adoption of whole-slide imaging (WSI), which is crucial for improving diagnostic workflows. DP has been shown to enhance efficiency by enabling faster access to slides, facilitating remote consultations, and reducing delays associated with traditional methods [18]. As noted by Fraggetta et al. [19], routine DP workflows streamline diagnoses by improving accessibility and enabling quicker decision-making, which ultimately leads to better patient outcomes. The integration of slide scanners and camera attachments in Romanian laboratories would help phase out glass slides, improving archiving, streamlining storage, and providing students and trainees with easier access to pathology digital slides for learning. Additionally, these tools could help optimize healthcare costs by reducing errors, improving resource management, and boosting efficiency across settings [20]. This gap in digital tools represents a missed opportunity to enhance diagnostic accuracy, workflow efficiency, and patient care. While this is not a complete digital transformation, implementing these basic tools would be an important first step toward modernizing pathology practices and improving overall healthcare delivery.

The importance of molecular pathology in cancer diagnosis and treatment has been increasingly recognized worldwide as being fundamental for optimal patient care. A review by Labisso et al. [21] on the status of PSs in Sub-Saharan Africa highlighted how crucial molecular diagnostic tools are for accurate cancer treatment. Similarly, our study highlights limited access to such technologies in Romania. Only 1.25% of the pathology laboratories surveyed have NGS, and just 7.5% have PCR capabilities, all of which are concentrated in larger city hospitals. This reflects a significant gap in access to advanced diagnostic tools, particularly for patients in rural areas or smaller healthcare facilities. 

Despite significant gaps in infrastructure and financial resources, the satisfaction levels reported by pathologists in our study were surprisingly high, with around 70% of respondents rating their satisfaction above 5 (out of 10). This apparent contradiction could be attributed to several factors. One possibility is that pathologists have become accustomed to the systemic limitations within the public HS, including the underappreciation of their specialty. Furthermore, the growing presence of private PLs in Romania may influence pathologists’ satisfaction, as they might feel a sense of relief knowing that higher-quality HSs are available in the private sector—albeit at a cost. In some cases, pathologists who work in both public hospitals and private laboratories may have a clear understanding of the limitations within the public system. This dual role can lead them to refer patients to private facilities, where they can access more advanced diagnostic resources that may not be available in the public sector. However, this situation raises an important concern: what about the people who cannot afford private healthcare? While some pathologists may feel that patients have access to improved services in the private sector, this shift could further exacerbate inequalities in healthcare access, particularly for lower-income populations who remain dependent on the often underfunded public system. Equally important is to mention that in all European countries, medical professionals, including both general practitioners and specialists, typically earn significantly more than the average wage across all industries. This trend is evident when comparing the salaries of surgical pathologists in Germany, Romania, and Bulgaria. In Germany, the average gross hourly rate for surgical pathologists is around EUR 90, while in Romania and Bulgaria, it is closer to EUR 35 [22]. These variations are influenced by factors such as the public or private sector, as well as the individual’s level of experience, with pathologists in the private sector earning more across all three countries. Therefore, in Romania, despite the relatively lower earnings compared to Germany, it is theoretically possible for pathologists to maintain a satisfactory standard of living, even though working conditions may not always meet modern standards [19,21]. 

One other important insight from this study is the need for enhanced collaboration among pathologists to foster improvements in the field. The process of identifying PSs and establishing contact proved to be more challenging than anticipated, with some professionals initially reluctant to engage. This highlights a broader issue of fragmentation within the profession, which can hinder collective efforts to drive progress. Moving forward, a stronger culture of collaboration will be essential for advancing the standardization of practices and ensuring the delivery of high-quality pathology services. By working together, pathologists can not only improve the efficiency and accuracy of their diagnostic work but also strengthen their collective voice in advocating for better resources, funding, and recognition from health authorities. While these challenges represent some of the limitations of the present study, they also point to opportunities for future growth and improvement within the field. 

We acknowledge the limitations of this study. Due to the survey-based methodology, we are limited by potential biases. The findings may not be representative of all the functioning public PLs in Romania. Furthermore, there is no accessible official registry of public PLs, despite efforts to obtain this information by reaching out to the National Institute of Public Health. This lack of access to centralized data made it challenging to compile a comprehensive list of laboratories for the survey. Additionally, some of the email addresses provided on hospital websites appeared to be outdated or no longer in use, further complicating the communication process and potentially limiting the response rate. Another limitation arose from an oversight in the survey design: we did not initially account for pathology services (PSs) that do not perform IHC or special stains, offering only routine H&E staining. As a result, some respondents contacted us separately to highlight this omission, which may have led to an incomplete representation of the full range of PLs available. Despite the mentioned limitations, our findings highlight the existing problems and difficulties that impede better workflow and more advanced patient care. Our limitations will be addressed in the future with more comprehensive and stratified studies.

## 5. Proposed Recommendations

*Increasing Funding and Resource Allocation* 

Boosting public investment in PLs is essential to address the persistent underfunding in lower GDP settings, improve infrastructure, and support the adoption of advanced diagnostic technologies. Aligning budgets with the actual costs of modern pathology can help set realistic benchmarks and drive more equitable healthcare access.

*Adopt and Enforce Minimum Equipment Standards* 

Implement and enforce the official guidelines on minimal equipment requirements to encourage a standardized level of diagnostic capabilities across laboratories. Regular audits and incentives for compliance can help ensure that all facilities meet at least the core instrument and reagent needs.

*Prioritize the Digital Transformation of Pathology* 

Equipping laboratories with slide scanners, cameras, and robust information technology systems expands capacity for remote consultations, speeds up turnaround times, and promotes digital archiving. Training personnel to use these technologies effectively can further enhance diagnostic accuracy and workflow efficiency.

*Expand Molecular Pathology Capacity* 

Investing in molecular diagnostic tools such as PCR and NGS increases the ability to detect and classify cancers accurately, enabling personalized treatment decisions that can significantly improve patient outcomes. Resource sharing and centralized testing hubs may ensure that smaller or rural laboratories can still access advanced testing.

*Strengthening Collaboration and Training* 

Fostering a culture of knowledge exchange among pathologists nationally and internationally helps drive innovation, standardize best practices, and bolster professional development. Expanding ongoing training opportunities, including digital strategies and emerging technologies, can empower pathology teams to adapt to evolving patient needs.

## 6. Conclusions

Ultimately, addressing the challenges within Romania’s HS necessitates systemic reforms that prioritize increased funding, more effective allocation of resources, and a focus on specialties like pathology, which have often been sidelined in modernization efforts. Reforming the training of pathology residents to align with technological advancements is essential, as is fostering greater collaboration between laboratories. Moreover, efforts to standardize diagnostic processes could significantly streamline the patient journey, reduce inefficiencies, and improve care outcomes. By focusing on these key areas, Romania can build a more responsive, equitable, and effective HS capable of meeting the evolving needs of both patients and healthcare professionals.

## Figures and Tables

**Table 1:** List of Mandatory Minimum Equipment for Pathological Anatomy and Forensic Services. Established by the Romanian Ministry of Health.

Equipment	Features
Automatic tissue cassette printer	
Storage cabinet (for slides and blocks)	
Microscopes with at least the following objectives	×4, ×10, ×20, ×40, ×100 (a microscope/pathologist), at least 25% of microscopes with an additional examination end
Automatic tissue processor	
Device for drying the plates/films that will be inserted into the electron microscope	
Microscope with at least the following objectives	×4, ×10, ×20, ×40, ×100 with immunofluorescence, polarized light, and digital camera with image processing
Thermostat	
Cryotome (or equivalent)	
Microscope with multiple examination heads (multi-head type)	with at least 4 additional examination heads and an indication arrow on the screen
Paraffin embedding station	with cooling and heating annexes
Electron transmission microscope	
Computers	minimum one computer/recorder, one computer/doctor and two/workspaces with at least one multifunctional/computer network
Microtome	
Slide scanner	
Hood with charcoal filter	
Sink or thermostatic bath	
Centrifuge	
Knifemaker	
Automatic IHC stainer	
Liquid-based cytology system	
Set of fine and ultra-fine pliers for gripping the grids	
Automatic stainer	
Manual staining kit	
Ultramicrotome	
Slide incubator for hybridization	
Polymerase chain reaction (PCR) or Real-Time PCR amplification	
Centrifuge for PCR plates	
Automated nucleic acid extraction equipment	
Nucleic acid amplification and detection equipment	Nucleic acid integration, extraction, amplification, and detection systems

## Data Availability

The data collected in the survey are available on request from the corresponding author.

## Supplementary Materials

The questionnaire can be downloaded at: https://www.mdpi.com/article/10.3390/healthcare13111302/s1. Assessment of the Current state of Pathology Services in Public Hospitals in Romania.

## Abbreviations

The following abbreviations are used in this manuscript:

PL	Pathology laboratories
HS	Healthcare system
AI	Artificial Intelligence
IHC	Immunohistochemistry
FISH	Fluorescence in situ hybridization
CISH	Chromogenic in situ hybridization
IF	Immunofluorescence
NGS	Next-generation sequencing
PCR	Polymerase chain reaction
EU	European Union
GDP	Gross Domestic Product
H&E	Hematoxylin and Eosin
DP	Digital pathology
WSI	Whole-slide imaging
PS	Pathology services
