# Supplementary material for: Survey-Based Insights into Romania’s Pathology Services: Charting the Path for Future Progress
Source: Healthcare (Basel). 2025 May 30;13(11):1302. doi: 10.3390/healthcare13111302 (PMC12155235; doi:10.3390/healthcare13111302)
Supplement: Supplementary file 1 [file healthcare-13-01302-s001.zip › healthcare-3602866-supplementary.pdf]

# Assessment of the Current state of Pathology Services in Public Hospitals in Romania

This form aims to assess the current state of Anatomical Pathology Services in Romania and has been developed to identify potential areas for improvement, serving as a foundation for alignment with European standards. The questionnaire includes 10 questions addressing aspects such as the level of equipment in your laboratories, the diagnostic techniques employed, the capability to digitally capture microscopy slides, the current state of infrastructure, and financial support.

*Please provide the name of the location where the Anatomical Pathology Service in which you work is based:*

1. **What types of equipment are available in your laboratory?**
   ☐ Tissue processor
   ☐ Embedding station
   ☐ Microtome
   ☐ Cryotome or cryostat for intraoperative (frozen section) examinations
   ☐ Automated stainer (autostainer)
   ☐ Manual stainer
   ☐ Immunohistochemistry autostainer
   ☐ Coverslipping machine
   ☐ Automated liquid-based cytology system

2. **What diagnostic techniques are used in your laboratory?**
   ☐ Immunohistochemistry
   ☐ Polymerase Chain Reaction (PCR)
   ☐ Immunofluorescence (IF)
   ☐ Fluorescence In Situ Hybridization (FISH)
   ☐ Chromogenic In Situ Hybridization (CISH)
   ☐ Next-Generation Sequencing (NGS)

3. **Do you have the capability to digitally capture microscopy slides?**
   ☐ Yes
   ☐ No

4. **If you answered "YES" to the previous question, in what form can these digital images be obtained?**

☐ Microscope-attached camera

☐ Slide scanner

☐ Other (please specify):

5. **What techniques do you consider necessary to implement in order to improve the functioning of your laboratory?**

6. **When was the last renovation of the Anatomical Pathology laboratory in which you work carried out?**
   ☐ Less than 3 years ago
   ☐ 3–5 years ago
   ☐ 5–10 years ago
   ☐ More than 10 years ago

7. **When was the last renovation of the Mortuary within the Anatomical Pathology Service in which you work carried out?**
   ☐ Less than 3 years ago
   ☐ 3–5 years ago
   ☐ 5–10 years ago
   ☐ More than 10 years ago

8. **How many senior physicians and/or specialists are employed in the Anatomical Pathology Service in which you work?**

9. **On a scale of 1 to 10, how satisfactory is the level of financial support for the Anatomical Pathology Service in which you work?**

1 2 3 4 5 6 7 8 9 10

(not satisfactory at all) (very satisfactory)

10. **On a scale of 1 to 10, how satisfied are you with the infrastructure of the Anatomical Pathology Service in which you work?**

1 2 3 4 5 6 7 8 9 10

(not satisfactory at all) (very satisfactory)
